# Author Correction: A cross-country study on the impact of governmental responses to the COVID-19 pandemic on perinatal mental health

**DOI:** 10.1038/s41598-023-30737-2

**Published:** 2023-03-03

**Authors:** Ana Mesquita, Raquel Costa, Rena Bina, Carmen Cadarso-Suárez, Francisco Gude, Carla Díaz-Louzao, Pelin Dikmen-Yildiz, Ana Osorio, Vera Mateus, Sara Domínguez-Salas, Eleni Vousoura, Drorit Levy, Samira Alfayumi-Zeadna, Claire A. Wilson, Yolanda Contreras-García, Mercedes Carrasco-Portiño, Sandra Saldivia, Andri Christoforou, Eleni Hadjigeorgiou, Ethel Felice, Rachel Buhagiar, Camellia Hancheva, Erilda Ajaz, Ana Uka, Emma Motrico

**Affiliations:** 1 School of Psychology, University of Minho, Campus Gualtar, 4710-057 Braga, Portugal; 2 EPIUnit - Instituto de Saúde Pública, Universidade do Porto, Rua das Taipas,n° 135, 4050-600 Porto, Portugal; 3 Laboratório para a Investigação Integrativa e Translacional em Saúde Populacional (ITR), Universidade do Porto, Rua das Taipas, n° 135, 4050-600 Porto, Portugal; 4 Hei-Lab: Digital Human-Environment Interaction Lab, Faculty of Psychology, Education and Sports, Lusófona University, Porto, Portugal; 5 School of Social Work, Bar Ilan University, Ramat Gan, Israel; 6 Department of Statistics, Mathematical Analysis, and Optimization, Group of Biostatistics and Biomedical Data Science, University of Santiago de Compostela, Santiago de Compostela, Spain; 7 Department of Epidemiology, Research Group On Epidemiology of Common Diseases, University Clinical Hospital of Santiago, Santiago de Compostela Health Research Institute (IDIS), Santiago de Compostela, Spain; 8 Department of Psychology, Kirklareli University, Kirklareli, Turkey; 9 Graduate Program on Developmental Disorders and Mackenzie Center for Research in Childhood and Adolescence, Center for Biological and Health Sciences, Mackenzie Presbyterian University, São Paulo, Brazil; 10 Psychology Department, Universidad Loyola Andalucia, Sevilla, Spain; 11 Department of Psychology, School of Philosophy, National and Kapodestrian University of Athens, Athens, Greece; 12 The Center for Women’s Health Studies and Promotion, Ben-Gurion University of the Negev, Beersheba, Israel; 13 Nursing Department, School of Health Sciences, Ashkelon Academic College, 78682 Ashkelon, Israel; 14 Section of Women’s Mental Health, King’s College London and South London and Maudsley NHS Foundation Trust, London, UK; 15 Department of Obstetrics and Puericulture. Faculty of Medicine, Universidad de Concepción, Concepción, Chile; 16 Department of Psychiatry and Mental Health. Faculty of Medicine, Universidad de Concepción, Concepción, Chile; 17 Department of Social and Behavioral Sciences, European University Cyprus, Engomi, Cyprus; 18 Department of Nursing, School of Health Science, Cyprus University of Technology, Limassol, Cyprus; 19 Department of Psychiatry, University of Malta, Msida, Malta; 20 Sofia University “St. Kliment Ochridski”, Sofia, Bulgaria; 21 Department of Education and English Language, Beder University College, Tirana, Albania; 22 Present Address: ProChild CoLAB, Campus de Azurém, Guimarães, Portugal

Correction to: *Scientific Reports* 10.1038/s41598-023-29300-w, published online 16 February 2023

In the original version of this Article Emma Motrico was incorrectly affiliated with ‘Department of Psychology, School of Philosophy, National and Kapodestrian University of Athens, Athens, Greece’. The correct affiliation is listed below.

Psychology Department, Universidad Loyola Andalucia, Sevilla, Spain

The original Article has been corrected.

